# Vanishing White Matter Disease in Children: An Unusual Association, a Novel Mutation, and a Literature Review

**DOI:** 10.7759/cureus.73667

**Published:** 2024-11-14

**Authors:** Zahra Alsahlawi, Hasan M Isa, Sulaiman Alresias, Sayed Mohamed Hasan, Husain A Malalla, Ayman K Ebrahim, Khadija Ali

**Affiliations:** 1 Department of Pediatrics, Arabian Gulf University, Manama, BHR; 2 Department of Pediatrics, Salmaniya Medical Complex, Manama, BHR; 3 Department of Neurology, Salmaniya Medical Complex, Manama, BHR

**Keywords:** bahrain, children, leukodystrophy, leukoencephalopathies leukoencephalopathy with vanishing white matter, novel mutation

## Abstract

Vanishing white matter (VWM) disease is an autosomal recessive disorder caused by mutations in the gene EIF2B encoding the subunits 1-5 of eukaryotic initiation factor 2B. Although rare, with a reported prevalence of 1:80,000 (0.001%), it was considered as one of the most common leukodystrophies. However, the worldwide incidence and prevalence of this disease are not clear.

In Bahrain, of 21 patients who were diagnosed with leukodystrophy, two patients were found to have VWM disease accounting for 9.5%. Vaccinations and infections were the trigger factors for this disease to manifest. Rapid neurological deterioration, loss of developmental milestones, and seizure disorders are the main presentations in both patients. Magnetic resonance imaging (MRI) showed the classical radiological changes of demyelination and leukodystrophy. Patient 1 had associated ulcerative colitis, a finding that was not reported before. Patient 1’s condition progressed to a vegetative stage, while patient 2 passed away, reflecting the poor disease outcome. In patient 2, a novel homozygous missense mutation was found in the EIF2B3 gene (c.25G>A, p.Ala9Thr).

In this report, we present in detail the prevalence of VWM disease among cases with leukodystrophy, patients’ characteristics, clinical presentations, radiological findings, associated diseases, genetic results, and clinical outcomes in the main tertiary hospital in Bahrain between 1998 and 2024. Moreover, we conducted a thorough literature review on this rare condition.

## Introduction

Vanishing white matter (VWM) disease (OMIM 603896) is one of the most common leukodystrophies [1,2]. It is an autosomal recessive disease caused by mutations in the gene EIF2B encoding the subunits 1-5 of eukaryotic initiation factor 2B [1-3]. The EIF2B gene is important for mRNA translation and regulation during the integrated stress response, and its mutations reduce the gene activity, as shown in the study by Abbink et al. [3]. In response to stressors of the EIF2B protein, which regulates protein synthesis, white matter demyelination can develop, leading to progressive leukodystrophy [3]. 

VWM disease was first recognized in 1994 by Schiffmann et al. [4]. It was described as “childhood ataxia with diffuse central nervous system hypomyelination” [3]. This disease can present in several clinical forms ranging from a prenatal, a subacute infantile (onset age <1 year), an early childhood (onset age 1 to <4 years), a late childhood (onset age 4 to <18 years), and an adult form (onset ≥18 years) [1]. The early childhood form is considered the most common type, while the infantile form is considered the most severe one [5,6].

Van der Knaap et al. proposed the following diagnostic criteria for VWM disease: (1) normal or mildly delayed initial motor and mental development; (2) a chronic progressive and episodic course neurologic deterioration that may follow minor infection and minor head trauma that may lead to lethargy or coma; (3) neurological signs including cerebellar ataxia, spasticity, optic atrophy, and epilepsy; and (4) a typical and symmetric involvement of the cerebral hemispheric white matter on magnetic resonance imaging (MRI) [7].

The worldwide incidence and prevalence of VWM disease are not clear despite the fact that it is considered a common type of leukodystrophy [1,5]. Moreover, in the Middle East, data about VWM disease are also scarce. Only a few cases were reported from the Arabian Gulf region. To our knowledge, one case was reported from Saudi Arabia in 2008 and another one from Kuwait in 2013 [5,8]. More recently, in March 2021, Alfadhel et al. published a spectrum of leukodystrophies in Saudi Arabia, including 11 patients with VWM [9]. However, no cases of VWM disease have been reported from Bahrain. In Bahrain, of 21 patients who were diagnosed with leukodystropy two patients were found to have VWN disease which account for 9.5% among this group of patients. Hence, this report aimed to review two patients diagnosed with VWM disease in Bahrain, along with a literature review.

## Case presentation

Case 1

This is a case of a seven-year-old Bahraini female who was a product of a full-term normal vaginal delivery to first-degree cousins. She had no history of perinatal complications. She was first seen in the pediatric outpatient clinic with complaints of limping and difficulty walking of a three-month duration that started immediately after receiving the 18-month vaccination (the second dose of measles-mumps-rubella; the fourth doses of diphtheria-pertussis-tetanus, *Haemophilus influenzae* type B, and oral polio vaccines). There was no history of fever or upper respiratory tract symptoms preceding her motor symptoms. The limping and frequent tripping were insidious in onset and were initially noted on the left side of the body, which progressed to involve both sides later.

**Figure 1:**
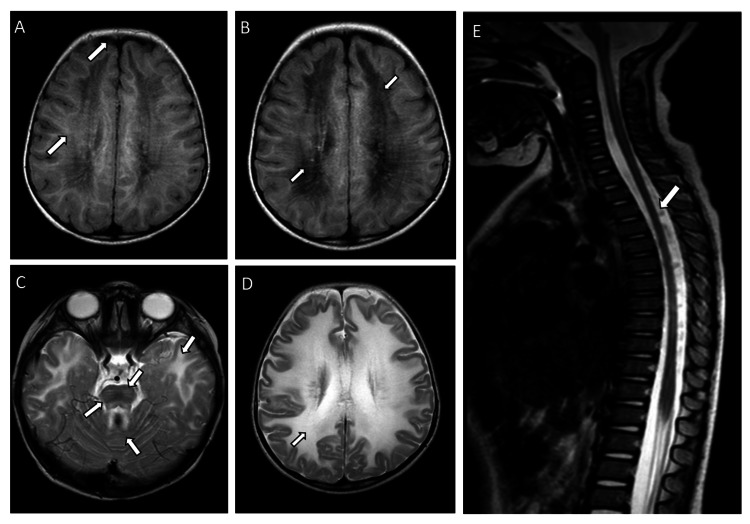
Axial flair brain and spine magnetic resonance imaging (MRI) of patient 1 at the level of the centrum semiovale (A) Brain MRI at the age of 21 months showing diffuse bilateral symmetrical periventricular white matter changes extending to the subcortical U fibers (arrows). (B, C) Repeated brain MRI at the age of two years showing further progression of the white matter disease with involvement of the brain stem. (D) T2-weighted brain MRI image showing the progression of the disease (arrow). (E) T2-weighted spinal cord MRI showing diffuse atrophic appearance (arrows).

On examination, the patient was conscious, alert, and interacting with her surroundings. There was no apparent involvement of cranial nerves. She had both appendicular and axial hypotonia with depressed deep tendon reflexes in both upper and lower limbs. The power was reduced to 4 out of 5. Otherwise, she did not show any signs of sensory defects or apparent cerebellar involvement. The patient started to be investigated in the neurology and metabolic clinics, and an MRI of the brain showed diffuse symmetrical periventricular bilateral white matter changes extending to the subcortical U fibers, giving it a tigroid appearance indicating leukodystrophy of VWM disease type (Figure 1A).

The diagnosis of VWM disease was confirmed by whole exome sequencing. A homozygous missense mutation was found in the EIF2B5 gene (c.318A>T, p.Leu106Phe). Both parents were heterozygous for the same mutation. The patient’s condition remained static up to the age of two years when she presented to the accident and emergency department with increased weakness and two attacks of partial complex seizures following an episode of upper respiratory tract infection. Her examination revealed intact consciousness and orientation, but she had marked deterioration in her motor functions in the form of generalized spasticity, with lower limbs being more affected than the upper limbs, hyperreflexia, and the upgoing Babinski sign. Her power reduced this time to 3 out of 5 in the upper limbs and 2 out of 5 in the lower limbs. Her sensory and cerebellar functions remained intact.

An EEG showed a slowing background with right temporal epileptiform discharges, for which she was started on oxcarbazepine. One day later, she developed a prolonged generalized tonic-clonic seizure, which did not respond to loading doses of phenytoin and phenobarbitone. Subsequently, she was shifted to the Pediatric Intensive Care Unit, where she was intubated and started on midazolam infusion, which eventually controlled her seizures. She was kept on regular phenytoin and levetiracetam until her EEG showed suppression. However, her overall neurological status started to deteriorate, and she was in a stupor state post-extubation. She was not fixing or following up and not responding to calls from parents. She lost her food swallowing skills, for which a nasogastric tube was inserted for feeding. She also became markedly spastic, and her muscle power reduced to 1 out of 5 in all limbs. Finally, she became in a vegetative state, quadriplegic, unable to maintain her upper airways, and ultimately tracheostomized and remained in the hospital for nursing care until the date of the study. A repeated MRI showed involvement of the spinal cord and brain stem with some thinning of the corpus callosum and internal capsule. There was a worsening of the white matter involvement in the form of generalized lysis with thread-like remnants (Figures 1B-1E). 

At the age of six years and while she was still in the hospital, she developed a fever and fresh bloody diarrhea. Her inflammatory markers were elevated (C-reactive proteins and erythrocyte sedimentation rate). She was given intravenous fluids and antibiotics. Her stool and blood cultures were sterile. Her hemoglobin level was 8.1 g/dL, and she received a blood transfusion. Abdomen ultrasound and Meckel’s diverticulum scintigraphy scan were negative. A colonoscopy showed continuous mucosal inflammation with multiple ulcers. Histopathology of colonic biopsies showed crypt branching, crypt abscesses, and cryptitis but no granulomas. Therefore, a diagnosis of ulcerative colitis was made, and she was started on prednisolone and mesalamine, for which bloody diarrhea improved.

Case 2

**Figure 2:**
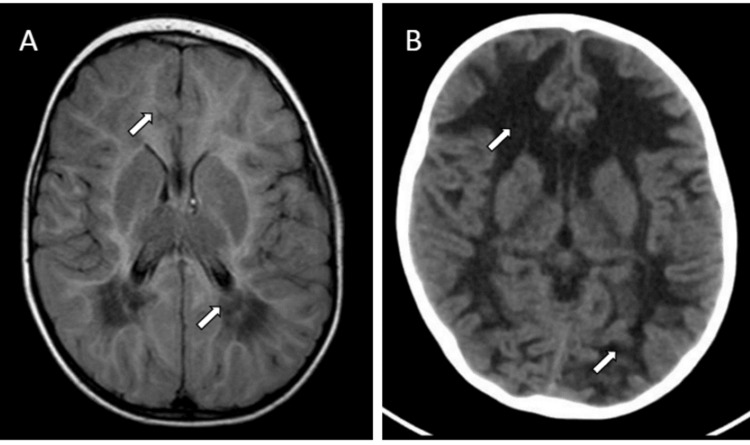
Brain imaging of patient 2 at the level of the centrum semiovale (A) Axial flair brain magnetic resonance imaging at the age of 17 months showing bilateral symmetrical diffuse confluent white matter abnormalities extending into the subcortical U fibers (upper arrow) with a high parietal tigroid appearance (lower arrow) in addition to the corpus callosum and internal capsule thinning. (B) A non-contrast axial brain computed tomography at the level of the basal ganglia, performed at the age of two years, showing further degeneration and total liquefaction of the white matter, which is more apparent in the frontal areas (upper arrow), in addition to generalized brain atrophy (lower arrow).

A 17-month-old girl was referred to the outpatient clinic with a history of a global regression of her developmental milestones following an upper respiratory tract infection. The patient was a product of full-term to non-consanguineous parents with no perinatal complications apart from a maternal urinary tract infection during the first trimester. Prior to this episode of sickness, the patient was thriving well and achieving developmental milestones properly, whereby she was cruising around furniture but not fully walking, saying mama and baba, and interacting well with her family members. After she had the episode of upper respiratory tract infection, she was not able to sit without support, she lost her ability to walk, and she lost her speech. On examination, the patient was conscious, alert, and crying when approached. Examination of the cranial nerves revealed no abnormalities. She was found to be generally spastic with hyperreflexia with a positive Babinski sign. She was investigated thoroughly, including metabolic workup, brain imaging, and genetic testing. MRI of the brain and spine showed bilateral symmetrical diffuse confluent white matter abnormalities extending into the subcortical U fibers with a high parietal tigroid appearance in addition to the corpus callosum and internal capsule thinning (Figure 2A).

These MRI findings are in line with the diagnosis of VWM that was confirmed by next-generation sequencing (NGS) for leukoencephalopathy genes. A homozygous missense mutation was found in the EIF2B3 gene (c.25G>A, p.Ala9Thr). The patient remained stable until she presented at the age of two years with multiple unprovoked tonic-clonic seizures. She was loaded with phenytoin and started on regular levetiracetam to control her seizures. EEG was found to be normal. A non-contrast axial CT brain showed further degeneration and total liquefaction of the white matter, which is more apparent in the frontal areas in addition to generalized brain atrophy (Figure 2B). One year later, she was admitted with fever and recurrent tonic-clonic seizures. On examination, she was in a vegetative state with axial and appendicular hypertonia, hyperreflexia, and scissoring of her lower limbs. Antiepileptic doses were adjusted. Her activity improved, but she did not go back to her baseline status as she lost awareness of her surroundings and was only decerebrating to painful stimuli. She also lost the ability to swallow, so she was started on nasogastric tube feeding. At the age of four years, she was again admitted with a status epilepticus, fever, and a history of choking. Her neurological examination was static compared to the last admission. Her chest examination revealed bilateral crepitations, but otherwise, the rest of the examination was unremarkable. Chest X-ray was suggestive of aspiration pneumonia, so she was kept nil by mouth and was started on clindamycin and cefotaxime. Her condition stabilized, and seizures were controlled. The CT brain findings did not change, but she continued to be oxygen-dependent. Later on, she was discharged on home oxygen. Three months later, she was admitted for fever and vomiting. A urine routine microscopy was suggestive of a urinary tract infection. She developed electrolyte imbalance with hypernatremia reaching 151 mmol/L and hypokalemia reaching 2.2 mmol/L. Subsequently, the patient had a sudden cardiac arrest with asystole. She passed away after full resuscitation and was declared dead at the age of four years and three months.

## Discussion

VWM disease, also known as “childhood ataxia with diffuse central nervous system hypomyelination” [3], is an autosomal recessive progressive leukodystrophy that is characterized by white matter demyelination in response to stressors caused by a mutation of the EIF2B protein, which regulates protein synthesis [3]. The worldwide incidence and prevalence of the disease are not available despite the fact that it is considered a common type of leukodystrophy [1]. Yet, a large longitudinal multicenter study from the Netherlands estimated the prevalence to be 1:80,000 cases per live birth based on reporting 33 Dutch patients out of 296 VWM patients referred from 198 centers worldwide [1].

Up to 2020, there were only two cases reported from the Persian Gulf region, one from Saudi Arabia and one from Kuwait [5,8]. In 2021, a relatively large study of the leukodystrophy spectrum was published in Saudi Arabia, and 11 more cases were reported [9]. One case was reported from Iran [10]. This rise in the number of reported cases is attributed to the emergence of whole exome and NGS.

The current study showed that loss of motor milestones after exposure to stressful conditions such as vaccination or upper respiratory tract infection were the key features in the cases of VWMD. Both patients in this study presented at the age of one and a half years that is going with the childhood-onset type of disease. This is similar to the age described in several studies published by Hamilton et al., Alfadhel et al., Güngör et al., and Turón-Viñas et al. [1,9,11,12]. This finding confirmed that childhood-onset VWMD is the most common category. Yet, two studies by Parvez et al. and Yavuz showed patients of younger age, i.e., three and four months of age, respectively [2,8].

The most common trigger identified for the disease was infections, as in patient 2 [1]. Vaccination is an occasional trigger and is considered an unusual presentation, which was the trigger in patient 1 [1]. Spasticity and hypotonia were reported in 82% and 52%, respectively, in the Dutch study [1]. These figures are comparable to the two patients in the current study as both of them had spasticity, while patient 1 was hypotonic during the attacks. Both patients in this study developed seizures. However, seizures were reported in 60% of 296 patients in the Dutch study [1]. Only 29% of patients reported in the Dutch study were bedridden, which is not the case in this study, as both patients were bedridden [1]. Moreover, 34% of their patients required assisted feeding, yet both patients in the current study required assisted feeding [1]. Patient 1 was diagnosed with ulcerative colitis, which was not reported in any literature.

In terms of radiological findings, MRI abnormalities are present in all affected individuals regardless of the age of onset and are even present in asymptomatic cases [7]. The typical MRI findings include diffuse symmetrical abnormal cerebral hemispheric white matter with signal intensity similar to or close to cerebrospinal fluid (CSF) in T1, T2, and flair images. Over time, the white matter will vanish and will be replaced with CSF [13]. In the present study, both patient 1 and patient 2 had similar MRI findings as their regional counterparts [5,8,9]. Diffuse white matter abnormality is present, involving the corpus callosum and the periventricular area. Likewise, two patients with the EIF2B3 mutation from Saudi Arabia had similar MRI findings [9]. Both of them showed low T1 signal intensities and high T2 intensities, confirming the presence of demyelination. In this study, patient 1 had a novel finding compared to the Middle East and the multicenter study from the Netherlands, with brainstem involvement seen on the MRI [1,9].

Both patients in the current study had a mutation detected by genetic testing. Patient 1 had a homozygous missense mutation found in the EIF2B5 gene (c.318A>T, p.Leu106Phe) based on the Human Genome Medical Database (HGMD) Professional 2020.1. [14]. This variant was first described as a disease-causing mutation for leukoencephalopathy with VWM disease by Leegwater et al. 2001 (PMID: 11704758) [15]. Several studies reported the EIF2B5 subunit as the most frequent location for VWMD gene mutation [1-2,11-12]. Patient 2 had a novel homozygous missense mutation found in the EIF2B3 gene (c.25G>A, p.Ala9Thr). The NGS panel of known leukoencephalopathy genes revealed this missense mutation, which was confirmed by conventional Sanger sequencing. To the best of our knowledge, this change has not been described in the literature or database so far, and nine of ten that used bioinformatic programs predict this alteration to be pathogenic (Table 1).

**Table 1:** Reported patients with vanishing white matter disease in Bahrain, neighboring countries, and worldwide *The present study NR, no record

Country	Author (year)	Patient (*n*)	Age	Trigger	Gene mutation (patient’s number)
Bahrain*	Alsahlawi et al. (2024)	2	1.5 years	Yes	EIF2B5 (n = 1), EIF2B3 (n = 1)
Saudi Arabia [9]	Alfadhel et al. (2021)	11	7 months to 2 years	NR	EIF2B4 (n = 7), EIF2B3 (n = 2), EIF2B2 (n = 2)
Kuwait [8]	Parvez et al. (2013)	1	4 months	Yes	EIF2B5 (n = 1)
Holland [1]	Hamilton et al. (2018)	296	3 years	Yes	EIF2B5 (n = 197), EIF2B2 (n = 49), EIF2B3 (n = 23), EIF2B4 (n = 22), EIF2B1 (n = 5)
Spain [12]	Turón-Viñas et al. (2014)	21	18 months to 8 years	Yes	EIF2B5 (n = 13), EIF2B3 (n = 2), EIF2B4 (n = 1)
Germany [11]	Güngör et al. (2020)	11	35 ± 19 months	Yes	EIF2B5 (n = 5), EIF2B3 (n = 3), EIF2B4 (n = 2), EIF2B1 (n = 1)
Taiwan [2]	Yavuz (2017)	2	3 months	No	EIF2B5 (n = 2)
Iran [10]	Khorrami et al. (2021)	1	7 years	No	EIF2B3 (n = 1)

Treatment of VWM disease is supportive, with no targeted therapy discovered to this date. A study done in 2019 showed a promising step in targeted therapy [3]. As the integrated stress response system is threatened with an EIF2B mutation, an experiment was conducted on biallelic mice with an integrated stress response inhibitor. This led to an increased expression of EIF2B, normalization of mRNA markers, and improvement in white matter pathology and motor skills [3].

Poor prognostic factors described in relation to VWMD were the episodic nature of the episodes, loss of ambulation, and seizures at an early onset [1]. Both patients in this study had all these factors.

This study is limited by the small number of patients, which is an expected issue when reporting rare metabolic and neurogenetic conditions such as VWM disease. However, the findings of this study are important as it is the first study from Bahrain to add new associations and report a novel mutation. Furthermore, this study is evolving the knowledge related to the clinical presentation and the underlying cause of this rare disease.

## Conclusions

VWM disease is a considerable type of leukodystrophy. Infections and vaccinations can trigger the disease’s neurological impairments and the development of classical radiological changes. A new disease association and a novel mutation were described in this study. The disease outcome is poor due to the lack of targeted therapy. Further studies are needed with a larger sample size and focusing on innovative therapeutic options.
